# Prevalence of Dementia Among the Elderly in Latin America and the Caribbean: A Systematic Review and Meta-Analysis

**DOI:** 10.1093/geroni/igab046.1855

**Published:** 2021-12-17

**Authors:** Fabiana Ribeiro, Ana Carolina Teixeira-Santos, Anja Leist

**Affiliations:** University of Luxembourg, Esch-sur-Alzette, Diekirch, Luxembourg

## Abstract

Background: Over the last decades, life expectancy in Latin America and the Caribbean showed a rapid increase, which led to a significant increase in the number of people with dementia. Moreover, 9% of the population in this part of the world are aged 65 or older, and by 2050 this percentage is projected to at least double. For this reason, it is essential to estimate the prevalence of dementia in LAC countries with the aim to determine suitable actions to enhance the quality of life of those affected. Methods: Database searches for articles were conducted September 2020 throughout Pubmed, Web of knowledge, Scopus, Lilacs, and SciELO. The inclusion criteria comprised population- or community-based studies, published in English, Spanish, or Portuguese, reporting data on the prevalence of dementia collected in LAC countries. The complete data search retrieved 1719 non-duplicates. Results: A total of 58 studies met the high-quality inclusion criteria, published 1991-2020, including participants in the following countries: Brazil, Mexico, Argentina, Colombia, Peru, Cuba, Dominican Republic, Venezuela, Ecuador, Trinidad and Tobago, and Jamaica. The most common form of dementia studied was Alzheimer’s disease with prevalence ranging from 5.9% to 23.4%. Estimates differed by age, gender, and education, with oldest, women, and lower-educated adults living in rural areas presenting higher dementia prevalence. Conclusion: This is the first study giving a comprehensive overview of dementia prevalence in LAC countries, which is relevant to estimate care needs and economic costs related to dementia treatment and care.

